# A clinical study to observe the efficacy and safety of Besunyen Detox Tea for constipation

**DOI:** 10.1097/MD.0000000000030729

**Published:** 2022-09-23

**Authors:** Wenting Fei, Jianjun Zhang, Linyuan Wang, Yi Yang, Yan Chen, Yawen Chen, Ran Tao, Yingli Zhu

**Affiliations:** a School of Chinese Materia Medica, Beijing University of Chinese Medicine, Beijing, China; b School of Traditional Chinese Medicine, Beijing University of Chinese Medicine, Beijing, China; c Conbio Technology Group Limited, Tianjin, China.

**Keywords:** functional constipation, health care, prospective observational study, real-world data, registry, syndrome differentiation and treatment, traditional Chinese medicine

## Abstract

Functional constipation (FC) is a common functional gastrointestinal disease and is one of the most common outpatient diseases. The purpose of this study was to investigate the efficacy and safety of the traditional Chinese medicine Besunyen Detox Tea (BDT) for FC and to compare the effect of BDT between constipation patients with and non-dryness-heat syndrome.

**Methods and analysis:**

This multicenter, prospective, observational registry study included 1000 participants diagnosed with FC. This study will collaborate with 3 comprehensive hospitals and 15 community hospitals and recruit patients into the registry between July 2022 and July 2023. After enrollment, we will collect the individual characteristics of each patient, anthropometric data and general condition, bowel movement, patient assessment of constipation symptoms, patient assessment of constipation quality of life, TCM syndrome scale, and time to take the laxative product again after treatment. We will also record adverse events and economic indicators at each visit.

**Discussion:**

This is the first registry-based study to collect real-world data of participants diagnosed with FC receiving BDT treatment. The results of this registry may also reflect these characteristics and provide direct clinical evidence to verify the importance of syndrome differentiation and treatment for the use of TCM health care products.

## 1. Introduction

Chronic constipation is a common disorder with a reported prevalence ranging from 3% to 27% in the general population.^[1,2]^ Its prevalence increases with age^[3,4]^ and consequently is expected to increase over the next few years.^[5]^ Functional constipation (FC) is a common functional gastrointestinal disease and one of the most common outpatient diseases. It refers to a group of independent clinical symptoms caused by non-organic causes, such as decreased frequency of defecation, difficult defecation, dry stool, and incomplete defecation, with a course of at least 6 months. FC can be classified into 3 categories: functional defecatory disorders, normal transit constipation, and slow-transit constipation.^[6]^ Different pathophysiological mechanisms may lead to FC, including abnormalities in the intestinal nervous system and neurotransmitters, dysfunction of cajal interstitial cells, abnormality of aquaporin, influence of intestinal flora, abnormal smooth muscle function, anxiety, and depression. Chronic constipation affects patients’ quality of life and can cause serious cardiovascular and cerebrovascular diseases.

The main treatment for FC is medication, including volume laxatives, irritant laxatives, prokinetic agents, osmotic laxative.^[7]^ In addition, colon hydrotherapy and biofeedback therapy can significantly improve the symptoms of patients with constipation.^[8]^ Traditional Chinese medicine (TCM) products have many advantages in health care and reduce the occurrence of chronic diseases. They have the characteristics of multicomponent, multitarget, and multichannel actions.^[9]^ However, there is not enough research to investigate the effectiveness of TCM health food, which is already in the market for the treatment of FC, and there is a lack of research on the characteristics of syndrome differentiation and treatment of TCM health products.

Besunyen Detox Tea (BDT) is a health tea used to improve gastrointestinal function, moisten the intestines, and relieve constipation among people with constipation for more than 20 years in China. BDT is a healthy Chinese tea consisting of six herbs, including Beishashen (Latin name: Glehniae Radix), Juemingzi (Cassiae Semen), Fanxieye (Sennae Folium), and Fuling (Poria Cocos (Schw.) Wolf.), Shanyao (Rhizoma Dioscoreae), and Lvcha (Camellia sinensis). Anthraquinone glycosides, free anthraquinones, and other polyphenols are the main components responsible for the laxative effect of Chinese herbal medicine.^[10]^

To investigate the efficacy and safety of traditional medicine BDT for FC, we conducted an observational registry study in the gastrointestinal clinic of 3 comprehensive hospitals and 15 community hospitals. To further compare the effect of BDT between constipation patients with dryness-heat syndrome and non-dryness-heat syndrome to reflect the characteristics of syndrome differentiation and treatment of TCM health care products.

## 2. Participants and Methods

### 2.1. Study design

**Figure 1.**
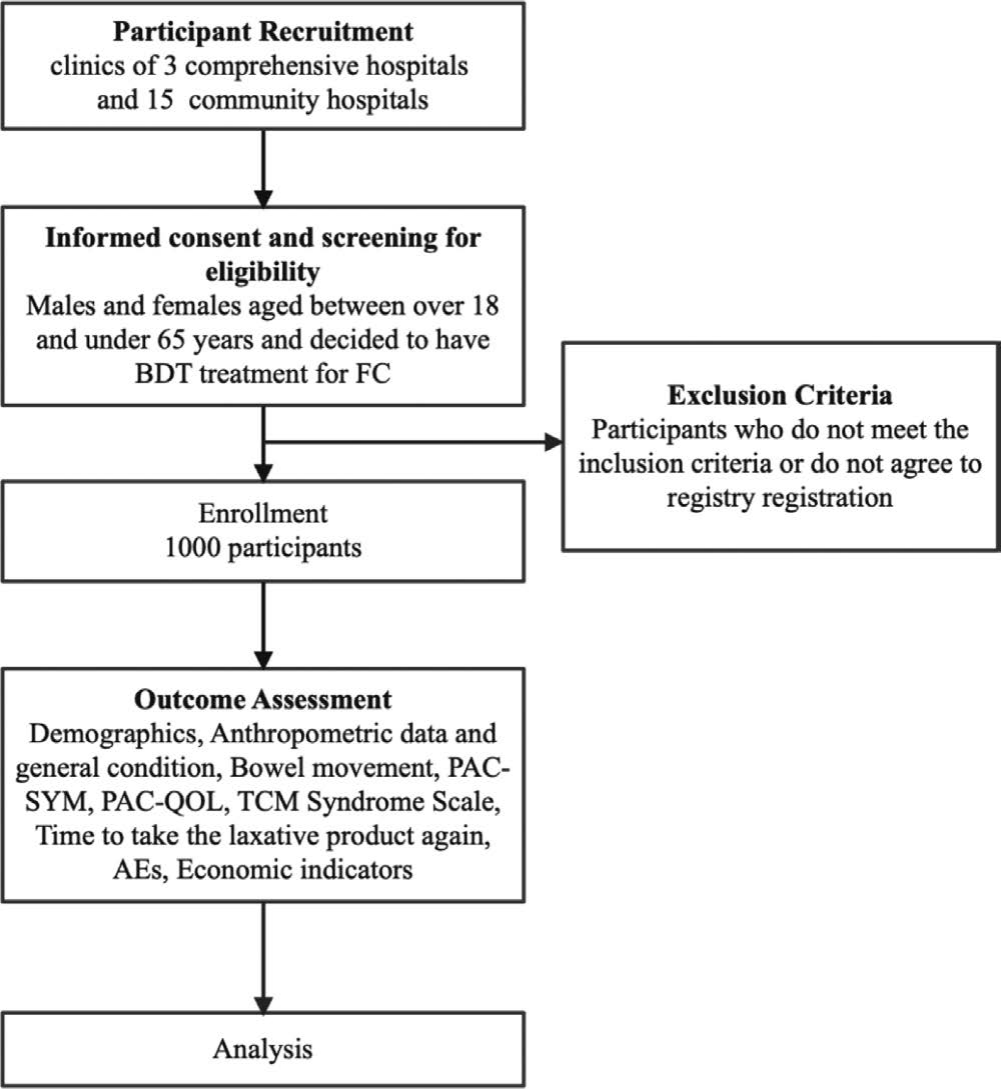
Study flow chart. BDT = Besunyen Detox Tea, FC = functional constipation, PAC-QOL = Patient Assessment of Constipation Quality of Life questionnaire, PAC-SYM = Patient Assessment of Constipation Symptoms questionnaire, TCM = traditional Chinese medicine.

The aim of the proposed study was to conduct a multicenter, prospective, observational registry study with 1000 participants who were diagnosed with FC (Fig. 1). The diagnosis of FC was based on the Rome IV criteria. Participants who voluntarily signed a written informed consent form after receiving sufficient explanation of the benefits and risks of participating in the study will be evaluated for compliance with the inclusion and exclusion criteria through a screening process on their first visit. After screening, eligible participants were assigned to the registry. BDT will undergo for 2 weeks and there will be 2 weeks of follow-up after the treatment. Participants could stop taking the BDT if FC was relieved after continuous treatment for at least 7 days.

The efficacy and safety of BDT in patients with FC has been accurately recorded under real conditions. The natural grouping method was used in this study, and the participants were not divided into groups or queues in advance. They will be divided into a dryness-heat syndrome group and a non-dryness-heat syndrome group by filling out the TCM syndrome scale when entering the study. The laxative efficacy of the two groups will be analyzed from different perspectives.

### 2.2. Trial registration

This study was registered with the Chinese Clinical Trial Registry (http://www.chictr.org.cn). (Registration No.: ChiCTR2200061535), and the registration was on June 28, 2022, version 3.0. URL of trial registry record: http://www.chictr.org.cn/showproj.aspx?proj=172303.

### 2.3. Eligibility criteria

#### 2.3.1. Inclusion criteria.

Eligible patients will be included according to the following criteria: Men and women aged between 18 and 65 years of age; Diagnosed of FC was diagnosed using Rome IV criteria. The Rome IV criteria include the following symptoms^[8]^: (a) straining, (b) hard stools (Bristol 1-2), (c) sensation of incomplete evacuation, (d) sensation of anorectal obstruction, (e) need for manual maneuvers to facilitate evacuation, and (f) less than three spontaneous bowel movements per week. These symptoms were present in 25% of patients; Not taken orally or used externally as health food and drugs (including dietary fiber and probiotics) with laxative effects in the past 4 weeks; Voluntary informed consent.

#### 2.3.2. Exclusion criteria.

Those with unclear complaints, those who were too weak to carry out the study, and those who could not eat orally; Constipation symptoms caused by surgery within 30 days; Acute gastrointestinal disease occurred within 30 days of admission; Menstruation, pregnancy preparation, pregnancy, and lactation; Patients with serious systemic diseases of the cardiovascular, liver, kidney, and hematopoietic systems; Constipation with abdominal pain (irritable bowel syndrome); Recent defecation difficulties caused by severe organic diseases (colon cancer, severe enteritis, intestinal obstruction, inflammatory bowel disease, etc); History of drug and food allergy or contraindications to the study product; Mental disorders and cognitive dysfunctions; There are other accompanying diseases under treatment; Health food and drugs (including dietary fiber and probiotics) with laxative effects will be taken orally and externally 4 weeks before the trial, which will affect the judgment of the results.

### 2.4. Recruitment

Participants will be recruited from 3 comprehensive hospitals and 15 primary medical institutions across China. We will also advertise through websites and post recruitment posters on the walls of the entrance of each clinic to encourage voluntary participation in this study. If the participants met the inclusion criteria in this study, the researcher will fully explain the aim of this study and details of the procedures, including research usage, confidentiality, and provision of data to third parties for analysis, and will obtain informed consent from the potential participants. Simultaneously, professional clinicians will be arranged to observe the status of the participants and provide medical assistance.

### 2.5. Exposure

The participants will receive treatment with BDT (Beijing Aote Shuer Health Care Product Development Co. Ltd, Lot Number: 01200703, Beijing, China) twice a day (1 hour after lunch and 1 hour after dinner). The specification of tea is 2.5g/bag, and it is brewed once with 150 to 250 mL of boiling water. Health food and drugs (including dietary fiber and probiotics) with laxative effects shall not be taken orally and externally 4 weeks before the trial and during the trail, which will affect the judgment of the results.

During the experiment, the diet, drinking water, exercise, and rest were basically the same as before. If the constipation is aggravated, it is recommended that the participant go to the hospital for physical examination and use other laxative drugs. In case of serious adverse reactions, the participant shall immediately stop taking this product and seek medical treatment in time. The trial unit will bear the treatment costs and corresponding compensation arising therefrom.

The compliance of the participants was monitored by the counting method of the test products. Compliance = (actual dose/expected dose) × 100%.

### 2.6. Outcomes measures

Researchers determined that the following data will be collected to observe the efficacy and safety of the TCM BDT for FC. The detailed research schedule for this registry study is presented in Table 1.

**Table 1:** Research schedule of the registry study.

	Enrollment baseline	Subsequent visits	End of treatment	Subsequent visits
		The 7th day		The 14th day after the end
Informed consent	√			
Eligibility screening	√			
Matters needing attention	√			
Demographic characteristics	√			
Physical examination form	√	√	√	√
Bowel movement dairy (everyday)	√	√	√	√
PAC-SYM	√	√	√	√
PAC-QOL	√	√	√	√
TCM Syndrome Scale*	√	√	√	√
Economic questionnaire			√	
AEs		√	√	√

Note: the time window is specified as 5 days.

AE = adverse events, PAC-QOL = Patient Assessment of Constipation Quality of Life questionnaire, PAC-SYM = Patient Assessment of Constipation Symptoms questionnaire, TCM = traditional Chinese medicine.

* Only for people with dryness-heat syndrome.

1. Demographic characteristics: Age, sex, occupation, educational background, and medical history were collected as the basic demographic information.
2. Anthropometric data and general condition: Vital signs such as blood pressure, body temperature, rhythm, and pulse rate will be collected as variables related to general health conditions. Data on mental condition, sleep condition, and diet will also be collected as a general condition. These messages will be completed in a physical examination form.
3. Bowel movement: Participants were required to record the bowel movement diary during the treatment period every day, which included frequency, feeling in complete evacuation, and form with Bristol stool scale (ranging from “separate hard lumps” to “watery”).
4. Patient Assessment of Constipation Symptoms questionnaire. A US survey showed that the most frequent symptoms of chronic constipation were straining, hard stools, abdominal discomfort, bloating, infrequent bowel movements, and feeling of incomplete evacuation after bowel movement.^[11]^ Therefore, it is important to have a careful history assessing the presence of these symptoms and their duration and progression, and the Patient Assessment of Constipation Symptoms questionnaire is recommended for the clinical evaluation of patients with constipation.
5. Patient Assessment of Constipation Quality of Life questionnaire.^[12]^ The scoring items were divided into 28 items, and each item was divided into 5 grades according to the degree, with 1, 2, 3, 4, and 5 points. The higher the score, the worse is the quality of life.
6. TCM syndrome scale (only for dryness-heat syndrome). It was used to determine whether the participant belonged to the TCM syndrome of dryness-heat at the beginning of the study. These participants were required to record their TCM syndrome scale again after 14 days of treatment.
7. Time taken for the laxative product to appear again after treatment.
8. Adverse events (AEs): In case of occurrence of any AE, we will immediately provide appropriate treatment to the participant according to the guidelines of the clinic and then observe the participant’s progress during the follow-up visit.
9. Economic indicators: Cost-utility analysis. The economic questionnaire must be completely completed at the end of the treatment.

### 2.7. Data management and quality control

Researchers participating in clinical research must have professional qualifications and abilities, which are determined after a qualification review. The registration observation form was designed according to the study plan and data collection was completed by the clinician. All the collected data were managed using a confidential online database.

To ensure the accuracy of the data, training was performed before the start of the trial. Researchers should have a full understanding of the clinical study protocol and specific connotations of each index. The description of the subjective symptoms of the participants should be objective and should not be induced or prompted. The specified objective indicators were checked according to the time and method specified in the plan. In addition, attention should be paid to the observation of adverse reactions or unexpected toxicity and side effects.

### 2.8. Sample size calculation

We plan to recruit at least 1000 patients during the study period. Empirically considering the trends of patients visiting each community hospital, we estimated that approximately 1000 participants will be enrolled (100 patients or more per year per clinic) for a year. The study started in July 2022, is currently ongoing, and will finish in July 2023.

### 2.9. Statistical analysis plan

All statistical analyses were conducted using two-tailed tests and the significance level was set at 5%. An analysis of covariance will be performed when it is necessary to control for underlying variables, such as demographic variables. The propensity score can effectively balance the distribution and composition of each characteristic variable between groups and evaluate the relationship or role between intervention measures and outcome variables. It is often used to control for confounding bias among non-randomized groups in observational studies. The generalized estimating equation has obvious advantages over other methods for evaluating propensity score values and is the most recommended algorithm among the current evaluation methods for propensity score values.^[13]^ The mean of other scoring data will be analyzed using the *t* test or the chi-square test. The incidence of AEs within the groups was also evaluated using the chi-squared test. For missing data, the last observation carried forward will be used through a case report form inspection to replace the missing value. All statistical analyses in this study were conducted using SPSS 20.0 (SPSS Inc., IBM Corp., Armonk, NY).

### 2.10. Ethics and dissemination

Ethical approval for this study (2022BZYLL0402) was provided by the Ethics Committee of the Beijing University of Chinese Medicine, Beijing, on April 28, 2022. Written informed consent was obtained from all participants before enrollment in the study. The results will be published in a peer-reviewed journal and disseminated electronically and in print regardless of the results.

### 2.11. Data and safety monitoring boards

According to the operation guide for the establishment and functions of data and Safety Monitoring Committee issued by the World Health Organization, an independent Data and Safety Monitoring Boards (DSMB) will be established in this study. The committee is composed of 7 experts including clinical, social researchers, biostatistics, and other relevant personnel outside the research team, and will be responsible for the data evaluation during the study period, and will be responsible for the important data questions; the safety data report shall be consulted and monitored. In the study of this project, DSMB conducts safety assessment on the accumulated data of the clinical study to ensure the safety of the participants, monitor the implementation of the study, including the enrollment, protocol violation and dropout of the overall and each center, baseline characteristics, accuracy, completeness and timeliness of the monitoring data, and monitor the compliance of the researchers and participants with the protocol.

The research group shall report the serious AE report judged by the investigator to be possibly related or related to the research intervention to the DSMB chairman by telephone or email, and submit the formal written report. All other AEs and serious AE reports shall be summarized and submitted to the DSMB chairman (every month). Relevant analysis reports include but are not limited to the following: list of serious AEs, AEs, violations or deviations from the protocol, research progress report, list of dropped participants, etc. After DSMB reviews the data, it can suggest to continue as planned, continue or terminate the trial after modifying the protocol, or suspend the study until more supporting information is available.

### 2.12. Informed consent

Before each participant is enrolled in this study, the investigator has the responsibility to fully and comprehensively introduce the purpose, procedure, and possible risks of this study to them in written form. Participants should be informed that they have the right to withdraw from the study at any time, and each patient must be given an informed consent notice sheet before enrollment. The investigator is responsible for ensuring that each participant signs the informed consent before entering the study and keeps it in the study file. The personal information about potential and enrolled participants will be protected confidentiality before, during, and after the trial.

## 3. Discussion

This paper describes the protocol of a multicenter, prospective, registry-based observational registry study to monitor the efficacy and safety of the TCM BDT for FC. BDT is a functional food for Chinese herbal tea bags, which has a laxative effect and has been commonly used among people with constipation for more than 20 years in China. In particular, the active ingredients of BDT, such as total anthraquinones, sennoside A, sennoside B, and rhein, are the main compounds of BDT in the treatment of constipation. Previous studies have demonstrated that anthraquinone, especially its dianthrone compounds, such as sennoside A and sennoside B, exert significant laxative effects in promoting gastrointestinal motility and relieving FC.^[14]^

There are a large number of TCM health foods in China, but the guiding principles for the use of TCM health foods are currently lacking. In order to maximize the effect of TCM health foods on the importance of the application of TCM syndrome differentiation to health products, we put the standard of syndrome differentiation into the design of this study. As we know, TCM has its own properties (such as hot or cold), and it is theoretically believed that BDT has a better effect on constipation patients with dryness-heat syndrome. We aimed to identify the most suitable syndrome or clinical manifestation of BDT in this study.

This study is the first registry-based study to collect real-world data of participants diagnosed with FC receiving BDT treatment in community hospitals. The results of this registry may demonstrate the acceptance of BDT in patients and evaluate the effectiveness and safety of BDT for FC. Second, the long-term effect of BDT on FC was evaluated by observing the time the participants used the laxative product within 2 weeks of the end of the trial. Furthermore, the laxative effect of BDT on people with different syndromes (dryness-heat syndrome and non-dryness-heat syndrome) was investigated. The factors that affect the efficacy of BDT are analyzed by multiple factors, such as the severity of constipation, age, sex, occupation, and medical history. Finally, we evaluate the economic value of BDT. This is the first study to evaluate the effectiveness of TCM health products that are already on the market for people with different syndromes. This study has great significance.

In an open clinical study, quality control has become an important aspect worthy of attention in the process of implementation, and the concept of quality control should be implemented in every process of the study.^[15]^ For example, the method of continuous selection should be adopted to reduce the selection bias in the selection of participants. Clinical research should be strictly controlled for data collection, management, and analyses. We can carry out training for clinicians and research coordinators who collect data, make full use of the Internet, and establish a research website. The clinical diagnoses, relevant variables, and results of all participants were standardized to minimize the error of the collected data. The central database can be established, the regularly uploaded data should be manually checked, and existing problems need to be fed back to researchers immediately. This research will be more valuable with strict quality control.

## 4. Conclusion

In conclusion, this protocol will provide details for investigators regarding BDT in health care for FC. The results of this registry may demonstrate the acceptance of BDT in patients and evaluate its effectiveness and safety.

## Acknowledgments

We gratefully acknowledge the contributions of all participants in this study and all Chinese medicine practitioners who will contribute to the study, but not the list of authors.

## Author contributions

LYW is the principal investigator and project leader. JJZ managed the trial progress. LYW, JJZ, and WTF designed the protocol. The WTF developed the protocol and helped draft the manuscript. YC, YWC, and RT contributed to promoting participant recruitment. YC, YWC, RT, YLZ, and YY promoted recruitment and management of the assessment of participants. LYW and JJZ revised the manuscript. All the authors have read and approved the final manuscript.

**Data curation:** Yan Chen, Yawen Chen, Ran Tao, Yi Yang.

**Formal analysis:** Yi Yang.

**Investigation:** Wenting Fei, Yi Yang.

**Methodology:** Jianjun Zhang

**Project administration:** Linyuan Wang.

**Supervision:** Yawen Chen.

**Validation:** Ran Tao.

**Visualization:** Yingli Zhu.

**Writing – original draft:** Wenting Fei.

**Writing – review & editing:** Yingli Zhu.

## References

[1] Schmidt FM Santos VL. Prevalence of constipation in the general adult population: an integrative review. J Wound Ostomy Continence Nurs. 2014;41:70–6; quiz E1. 24378694 10.1097/01.WON.0000438019.21229.b7

[2] Mugie SM Benninga MA Di Lorenzo C. Epidemiology of constipation in children and adults: a systematic review. Best Pract Res Clin Gastroenterol. 2011;25:3–18. 21382575 10.1016/j.bpg.2010.12.010

[3] Talley NJ Jones M Nuyts G. Risk factors for chronic constipation based on a general practice sample. Am J Gastroenterol. 2003;98:1107–11. 12809835 10.1111/j.1572-0241.2003.07465.x

[4] Chiarelli P Brown W McElduff P. Constipation in Australian women: prevalence and associated factors. Int Urogynecol J Pelvic Floor Dysfunct. 2000;11:71–8. 10805262 10.1007/s001920050073

[5] Tack J Müller-Lissner S Stanghellini V. Diagnosis and treatment of chronic constipation – a European perspective. Neurogastroenterol Motil. 2011;23:697–710. 21605282 10.1111/j.1365-2982.2011.01709.x PMC3170709

[6] Lembo A Camilleri M. Chronic constipation. N Engl J Med. 2003;349:1360–8. 14523145 10.1056/NEJMra020995

[7] Vriesman MH Koppen IJN Camilleri M. Management of functional constipation in children and adults. Nat Rev Gastroenterol Hepatol. 2020;17:21–39. 31690829 10.1038/s41575-019-0222-y

[8] Aziz I Whitehead WE Palsson OS. An approach to the diagnosis and management of Rome IV functional disorders of chronic constipation. Expert Rev Gastroenterol Hepatol. 2020;14:39–46. 31893959 10.1080/17474124.2020.1708718

[9] Wang Y Fan X Qu H. Strategies and techniques for multi-component drug design from medicinal herbs and traditional Chinese medicine. Curr Top Med Chem. 2012;12:1356–62. 22690682 10.2174/156802612801319034

[10] Ma Q Wang CZ Sawadogo WR. Herbal medicines for constipation and phytochemical comparison of active components. Am J Chin Med. 2022;50:723–32. 35331086 10.1142/S0192415X2250029X

[11] Johanson JF Kralstein J. Chronic constipation: a survey of the patient perspective: patient perspective on constipation. Aliment Pharmacol Ther. 2007;25:599–608. 17305761 10.1111/j.1365-2036.2006.03238.x

[12] Marquis P De La Loge C Dubois D. Development and validation of the patient assessment of constipation quality of life questionnaire. Scand J Gastroenterol. 2005;40:540–51. 16036506 10.1080/00365520510012208

[13] Loke YK Mattishent K. Propensity score methods in real-world epidemiology: a practical guide for first-time users. Diabetes Obes Metab. 2020;22(Suppl 3):13–20. 32250525 10.1111/dom.13926

[14] Li Y Jiang JG. Health functions and structure-activity relationships of natural anthraquinones from plants. Food Funct. 2018;9:6063–80. 30484455 10.1039/c8fo01569d

[15] Kim HS Lee S Kim JH. Real-world evidence versus randomized controlled trial: clinical research based on electronic medical records. J Korean Med Sci. 2018;33:e213. 30127705 10.3346/jkms.2018.33.e213 PMC6097073

